# Modeling cuttlefish behavioural chromatophore response

**DOI:** 10.1186/1471-2202-13-S1-P4

**Published:** 2012-07-16

**Authors:** Zach Zboch, James Peterson

**Affiliations:** 1 Department of Biological Sciences, Clemson University, Clemson, SC 29634, USA

## 

**Figure 1:**
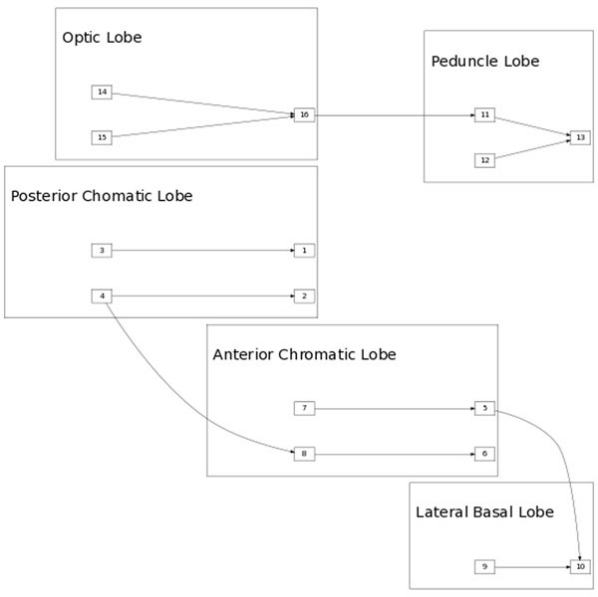
Cuttlefish chromatophore processing

We build a model of cuttlefish chromatophore excitation patterns due to environmental input. Specific inputs generate scripted chromtophore pattern responses on the surface of the skin. More complicated responses are then assembled from these templates as sequences of inputs arrive. We build a simplistic cuttlefish brain using neural architecture modeling tools written in MatLab which allow us to construct the brain model from individual modules using a vector addressing scheme [1] (Figure 1).

Inputs come into the sensory input module and are processed by the cuttlefish brain architecture into signals sent to the output module. The signals activate the individual chromatophores in the usual way giving an essentially binary on/ off pixel response. Different cues in the environment are mapped to known cuttlefish pigmentation overlays on the surface of the skin. Individual simple cues result in coarse patterns of chromotophore activation and upon receiving a sequence of simple cues, more complicated responses are constructed in a hierarchical fashion [1].

The chromatophore visibility is known to be due to a brain signal which when received energizes a ring of muscle which contracts and pushes a dot of ink up to the surface of the skin. Hence, activation signals lead to visible dots and lack of activation signal can be inferred from the loss of the pigment dot. Our model therefore treats each chromatophore as a binary switch which moves from 1 to 0 or vice versa depending on the signal sent to the output module from our cuttlefish brain processing [3,4].
